# Regression of bovine cutaneous papillomas via ivermectin-induced immunostimulant and oxidative stress

**DOI:** 10.5455/javar.2021.h525

**Published:** 2021-07-20

**Authors:** AbdulRahman A. Saied

**Affiliations:** 1 Touristic Activities and Interior Offices Sector, Ministry of Tourism and Antiquities, Aswan, Egypt; 2 National Food Safety Authority NFSA, Aswan, Egypt

**Keywords:** Bovine cutaneous papillomas, Regression, Oxidative stress, Histopathology, Ivermectin, Efficacy

## Abstract

**Objective:**

Ivermectin (IVM) could be used effectively to treat bovine cutaneous papillomatosis, a widespread viral skin disease that causes major economic losses in cattle. This study aimed to evaluate the regression of bovine cutaneous papillomas induced by IVM by estimating oxidative stress markers, besides clinicopathological and hematological findings.

**Materials and Methods:**

Twenty naturally infected animals with cutaneous papillomatosis were chosen randomly and diagnosed clinically and histopathologically. All the infected animals were divided into groups: Group I (*n* = 10), which received no treatment and was considered the control group. In Group II (*n* = 10), the animals were subcutaneously injected at 0.2 mg/kg of IVM 2 weeks apart during the 90-day experimental period. Papilloma regression was tracked clinically, papilloma biopsies were taken for histopathological analysis, and blood samples were taken for hematological and oxidative parameter testing.

**Results:**

From the 15th to 45th day after receiving IVM, papillomas began to fade. Necrotic areas, ulcerations, and lymphocytic infiltration were found in the histopathological studies, besides a decrease in papilloma epidermal proliferation. total erythrocytes count, packed cell volume, total leucocytes count, and lymphocytes values were increased significantly, and a large decrease in glutathione peroxidase and glutathione reduced was identified as signs of IVM-induced oxidative stress.

**Conclusion:**

IVM has oxidative and immunostimulatory properties, and it can be used against cutaneous papillomatosis.

## Introduction

Ivermectin (IVM) is one member of the class of macrocyclic lactones that has been widely used in human and veterinary medicine as a broad-spectrum antiparasitic drug. Some researchers used IVM for new medical applications as an antiviral and anticancer agent [1,2]. Many studies have recorded the therapeutic impact of IVM against bovine papillomatosis (BP) [3–5], focusing only on the clinical evaluation of the IVM effect through papillomas’ regression monitoring.

Skin diseases are considered one of the most common causes of animal owners taking their animals to the veterinarian. To the best of our knowledge, there is no proof of IVMs anti-papilloma activity based on *in vitro* studies. In Egypt, IVM is widely used to treat cutaneous warts. Still, no clinical trials have been carried out to verify the therapeutic efficacy of this commonly used drug as we conducted in this study. 

BP is a common viral skin disease in cattle, affecting mainly young animals up to 2 years of age. Bovine papilloma viruses (BPVs) are the causative agents of BP and other tumors and cancers in different body regions such as the urinary bladder and esophagus. BPV infection is more common in cattle than in other animals, resulting in significant economic losses in animal husbandry due to weight loss, retarded growth, deteriorated hide quality, lower milk and meat yields. BPV-induced multiple papillomas can regress spontaneously or progress to cancer [6]. There is a significant link between the development of warts and immunity as the immune response of cattle against BPV is poor.

Besides the clinicopathological and hematological evaluations [7,8], this study aimed to explore the correlation of IVM-induced oxidative stress and cutaneous bovine papillomas regression through the estimation of oxidative stress markers; malondialdehyde (MDA), glutathione reduced (GSH), glutathione peroxidase (GSH-Px), and superoxide dismutase (SOD) [9]. MDA, GSH, GSH-Px, and SOD were estimated in the plasma and erythrocytes of papillomatosis-infected animals with or without treatment administration. Cattle infected with cutaneous papillomatosis were diagnosed based on morphological characteristics and histopathological features.

## Materials and Methods

### Ethics statement

In this study, infected animals were randomly selected after the owners gave their oral consent for sampling, treatment regimen, and photography. Every effort was made to keep the animals as pain-free as possible, and all procedures were carried out and approved by the Ethical Committee for the Use and Welfare of Experimental Animals at Aswan University, Egypt (Approval no.: ASWU2000017).

### Drugs and chemicals

Alfamec^®^ 1% was purchased from Alfasan Co., Woerden, The Netherlands; and the formula used for cattle and sheep injection was 1 ml Alfamec contains 10 mg IVM. The hematoxylin and eosin stain was procured from Sigma Chemical Co., Saint Louis, MO. Ethanol was purchased from Loba Chemie Co.; MDA, SOD, GSH, and GSH-Px Kits were procured from Biodiagnostic (Diagnostic and Research Reagents) Company, Dokki, Giza, Egypt. All other chemicals were of analytical grade and locally purchased.

### Study design

The study was conducted on client-owned native cattle located in Aswan, Upper Egypt. Twenty native Egyptian cattle *Bos taurus* (14 females and 6 males) exhibiting cutaneous papillomatosis were randomly chosen for sample collection. The age of study animals ranged from 11 months to 2.2 years and weighed 120–280 kg with unlimited access to food and water. All animals were clinically diagnosed and histopathologically confirmed. Animals’ sex, age, and weight were registered. Animals were categorized into two groups: Group I (*n* = 10) in which the animals received no treatment and were considered as control and Group II (*n* = 10) in which the animals were subcutaneously injected at 0.2 mg/kg of IVM (Alfamec^®^, Alfasan Co., Utrecht, Netherlands) 2 weeks apart. The efficacy was determined by wart count reduction test and post-treatment histopathological observations. Additionally, the hematological and oxidative stress parameters were assessed as well.

### Clinicopathological evaluation

The animals were monitored biweekly for up to 3 months after the treatment regimen began. IVM efficacy was measured by counting regressed warts and time elapsed for infected animals to heal compared with the control group.

Fourteen days post-treatment, biopsies were taken from different sites and collected by a specialized veterinary surgeon. Papillomas specimens were excised, collected, and fixed in 10% formalin. Specimens were dehydrated, cleared, and embedded in paraffin for sectioning at a thickness of 5 μm. Sections were subsequently stained with hematoxylin and eosin (H&E) stain. The slides were then observed and analyzed under a light microscope.

### Hematological profile

Blood samples were obtained from the animals’ jugular veins 14 days post-treatment in both groups [9–11]. For complete blood count [red blood cells count (RBCs); hemoglobin concentration (Hb); packed cell volume (PCV); total leucocytes count (TLC), and lymphocytes (L)], blood samples were collected in EDTA-containing tubes and all hematological procedures were carried out on a hematology analyzer.

### Oxidative stress biomarkers analysis

Blood samples were collected in EDTA-containing tubes and centrifuged for 15 min at 1,000 rpm to separate plasma and erythrocytes for biochemical assays and stored at −70*°*C for processing. After centrifugation, the buffy coat was removed, and the plasma and erythrocytes were washed with physiological saline. The erythrocytes were hemolyzed by vigorous vortexing. SOD [12], GSH-Px [13], GSH [14], and MDA [15] were measured, and all procedures were carried out according to the manufacturers’ instructions. All four parameters were measured spectrophotometrically (Shanghai Aucy Scientific Instrument Co., Ltd., Shanghai, China).

### Determination of MDA

Plasma was mixed well with chromogen; test tubes were covered with glass beads and then put in a hot water bath at 95*°*C for 30 min. After cooling, the optical densities were measured spectrophotometrically at 534 nm to assess the changes in MDA levels in plasma samples, and the results were expressed as nmol/ml.

### Determination of SOD

The washed centrifuged erythrocytes were mixed with cold redistilled water and left to stand at 4*°*C for 15 min, and the lysate was stored at −70*°*C for estimation of SOD level. Nitroblue tetrazolium and lysate were mixed well and then phenazine methosulfate was added to initiate the reaction. SOD activity in hemolysate was measured spectrophotometrically at 560 nm. SOD activity was expressed in unit/ml. 

### Determination of GSH-Px

GSH-Px activity in the sample was measured according to Paglia and Valentine’s method [13]. The activity of GSH-Px was measured at 340 nm by measuring the decrease of NADPH absorbance using an extension coefficient of 6.22 mM^−^^1^ cm^−1^, and the results were expressed as mU/ml. After harvesting the RBC, erythrocytes were washed three times with four volumes of normal saline. The red cell pellet was lysed by adding four volumes of cold distilled water to the estimated volume, centrifuging at 4,000 rpm for 10 min for collecting supernatant, then stored at −70*°*C for estimation of GSH-Px level.

### Determination of GSH

The erythrocytes lysate was centrifuged at 4,000 rpm for 10 min for collecting supernatant then stored at −70*°*C for estimation of GSH level. The lysate was mixed well with trichloroacetic acid, allowed to stand for 5 min, and centrifuged. The supernatant was mixed well with DTNB and measured after 5–10 min at 405 nm, and the results were expressed as mg/dl.

### Data analysis

The study results were presented as mean values ± standard deviations. One-way analysis of variance and paired *t*-test were used to statistically analyze the obtained data using computerized Statistical Package for the Social Sciences (statistics Ver. 16) software. Statistical significance was assumed at the *p* < 0.05 level.

## Results

### Clinicopathological findings

**Figure 1.**
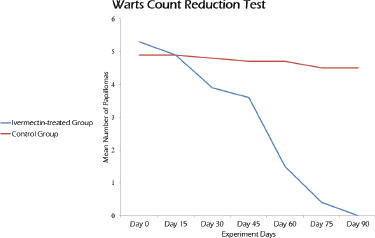
Wart count reduction test for the IVM-treated cattle infected with cutaneous papillomatosis in comparison with the untreated group.

**Figure 2.**
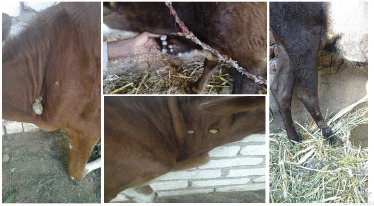
Gross appearance of papillomas with varying diameters pedunculated and rice grain-shaped present in the neck, dewlap and leg of different animals.

**Figure 3.**
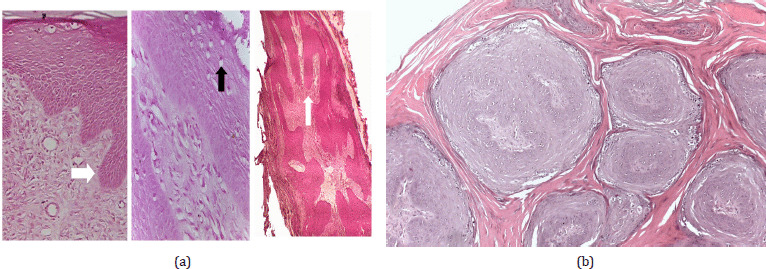
(A) Pre-treatment biopsies revealing finger-like projections (white arrow) with hyperplasia of stratum spinosum of epidermis and hyperkeratosis supported by fibroblastic proliferation of connective tissue core. Presence of koilocytes (keratinocytes within the stratum spinosum with a clear perinuclear halo) (black arrow). (B) Multiple papillomas showing islands of epithelial cells surrounded by lamellar keratin (H&E stain).

For 3 months, treated and untreated animals were monitored, and wart count reduction test (Fig. 1) was used to determine IVM efficacy. In both groups, animals were clinically (Fig. 2), and histopathologically (Fig. 3) diagnosed with cutaneous papillomatosis.

**Figure 4.**
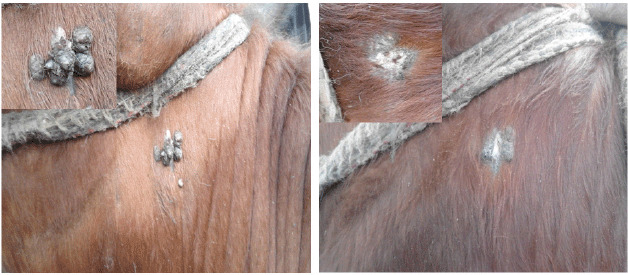
Two-year-old pregnant cow with round and dry papillomas at the neck (left). The same case after 60 days post-treatment showed complete disappearance of papillomas (right).

**Figure 5.**
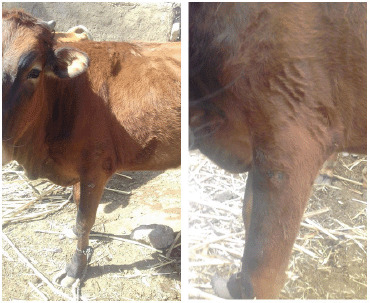
Eleven-month-old calf exhibiting dry, round, and cauliflower lesions, size 2–3 cm, on the left forelimb (left). The same case after 90 days post-treatment showed complete regression and skin healing (right).

Papillomas began to disappear from the 15th to the 45th day post-treatment, while the full cure was noted within the 30th–90th day post-treatment (Figs. 4 and 5). The control group showed nearly no changes in the number of warts. Pre-treatment biopsies in the treated group, besides the biopsies of the control group before and after the experiment, showed multiple finger-like projections with hyperplasia of stratum spinosum of the epidermis hyperkeratosis and acanthosis (Fig. 3), indicating the typical form of papillomatosis. 

**Figure 6.**
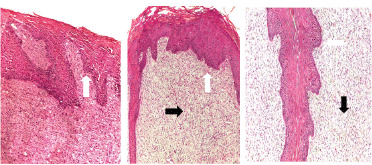
Post-treatment biopsies showing a decreased epidermal proliferation and an increased fibroplasia of connective tissue core (blunt finger-like projection) (white arrow). The dermal layer was infiltrated with lymphocytic cells (black arrow) (H&E stain).

**Figure 7.**
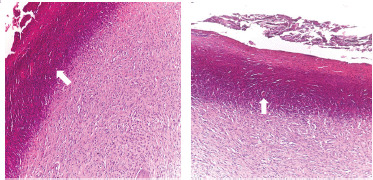
Ulceration covering epithelium of papilloma (white arrow) was observed with fibrosis of underlying tissue with granulation tissue formation rich in neovascularization and fibroblasts.

Post-treatment biopsies in the IVM-treated group revealed a regression in warts where the epidermal proliferation decreased and became a thin layer, while the connective tissue core showed fibroplasia (Fig. 6). Tissue necrosis with many fibroblast cells and infiltration of inflammatory cells, mainly lymphocytes (Fig. 6), and ulceration of covering epithelium of papilloma (Fig. 7) were observed.

### Hematological findings

Previous studies reported alterations in hematological parameters in animals infected with cutaneous papillomatosis compared with healthy animals. In the IVM-treated animals, RBCs count, PCV%, TLC, and lymphocytes percentage were significantly increased (*p* ≤ 0.05) in comparison with animals of the control group (Table 1). 

### Oxidative stress makers

In this study, erythrocytes were selected to quantify the antioxidant enzyme activities in treated and non-treated animals due to the ease of obtaining erythrocytes and based on previous papers that reported the presence of general oxidative stress in animals and humans infected with cutaneous papillomatosis [16,17]. 

The IVM-treated animals exhibited an insignificant increase in MDA (3.9 ± 0.4: 4.97 ± 0.3), while GSH (5.2 ± 0.3: 4.5 ± 0.1) and GSH-Px (77.03 ± 1.2: 37.15 ± 0.78) activities significantly decreased when compared with the control (untreated) animals (Table 2). Reduced GSH and GSH-Px activities (antioxidant enzymes) and increased MDA level (oxidative stress marker) in the IVM-treated animals indicated development of oxidative stress following IVM treatment and its possible role in papilloma regression.

## Discussion

IVM is one of the most influential and widely used anti-parasitic agents with a broad-spectrum activity against a wide range of numerous endoparasites and ectoparasites, especially nematodes and arthropods [18] in humans and animals. Avermectins have antibiotic and anti-tumor activities [1]. Moreover, IVM has an immunomodulating activity through enhancing immune cells [3], as well as strong antiviral [19,20] and anticancer [21] activities. All these activities were experimentally reported *in vitro*. 

Cutaneous BP is a common viral disease caused by BPV, which can regress or progress to malignancy, resulting in catastrophic economic losses in cattle [22]. Cutaneous papillomatosis in cattle and camels [8,23] is always associated with oxidative stress estimated from animal plasma and erythrocytes. The increased level of plasma MDA and oxidative stress in cattle with papillomatosis may suppress the reactive oxygen derivatives to the T-lymphocytes [24]. Therefore, enhanced antioxidant capacity and decreased oxidative stress improve animal treatment rates [9]. Although various methods have been used to treat bovine papillomas, effective medicines for this ailment are yet to be available.

**Table 1.** Hematological profile parameters in control and IVM-treated groups (mean ± SE).

Hematological parameters	Control group (*n* = 10)	IVM-treated group (*n* = 10)
RBCs (×10^6^/mm^3^)	6.52 ± 0.18	7.24 ± 0.13*
Hb (g/dl)	7.56 ±.15	9.33 ± 0.3
PCV (%)	32.2 ± 0.9	40.2 ± 0.41*
TLC (×10^3^/mm^3^)	11.05 ± 2	16.2 ± 1.1*
Lymphocytes (%)	48.62 ± 0.74	68.3 ± 0.63*

* Significant difference between both groups when *p* < 0.05.

**Table 2.** Oxidative stress markers in control and IVM-treated groups (mean ± SE).

Oxidative stress markers	Control group (*n* = 10)	IVM-treated group(*n* = 10)
MDA (nmol/l)	3.9 ± 0.4	4.97 ± 0.3
SOD (U/ml)	3.6 ± 0.2	3.4 ± 0.1
GSH-Px (mU/ml)	77.03 ± 1.2	37.15 ± 0.78*
GSH (mg/dl)	5.2 ± 0.3	4.5 ± 0.1*

* Significant difference between both groups when *p* < 0.05.

When IVM is subcutaneously injected, it will be distributed from the plasma to the skin [25,26] of healthy cattle, and repeated doses increase the drug concentration in the skin. Also, IVM reaches the stratum corneum via the sebaceous glands [27], where its effect on cutaneous BP.

In our study, in the IVM-treated animals, all papillomas disappeared at different times during the field trial with full efficacy, as evidenced by the histopathological studies, which revealed a decrease in epidermal proliferation with necrosis of dermal cells, and lymphocytic infiltration suggesting the antitumor and the immunostimulant effects of IVM. Other studies reported that IVM kills breast cancer cells through a mixed apoptotic and necrotic mechanism [28].

Previous studies found that cutaneous papillomatosis in camels and cattle resulted in substantial alterations in the hematological parameters, including a significant decrease in total erythrocytes count, Hb concentration, and PCV [8,23]. The development of anemia associated with papillomatosis may be attributed to the marked off-food as disease sequelae or due to the variety of cytokines involved in the chronic inflammatory process [29]. BP causes neutropenia, lymphopenia, and consequently, the development of leucopenia due to its action to form the circulating leucocytes and/or release endogenous corticosteroids in response to stress [30]. Our study reported an improvement in the blood picture of the IVM-treated animals. A significant elevation in RBCs count and PCV% showed that it could be due to increased appetite due to rapid healing of lesions. Animals became calmer, and their food intake increased, resulting in improvement of the body condition. Our findings agreed with Ahammed et al. [31] who evaluated the effect of IVM on hematological parameters when used against gastrointestinal nematodes and ectoparasites in cattle.

We found a significant increase in TLC and lymphocytes, which could be attributed to IVM’s effect on the animal’s cellular immune response, as previously reported [32]. Wart regression or healing could have occurred due to the immunomodulatory and antitumor effects of IVM [33].

Oxidative stress mechanisms were involved in the development of cutaneous diseases and disorders [34]. Excess accumulation of reactive oxygen species (ROS) causes lipid peroxidation and subsequently pathological changes in the cells, tissue function, and inducing skin carcinogenesis [35,36]. Antioxidant imbalance plays a pivotal role in the pathogenesis of skin diseases. MDA is recognized as a lipid peroxidation marker, and excessive MDA production has been associated with cellular injury in animals [37] and different pathological states [38].

MDA may be a more sensitive indicator for cutaneous papilloma in cattle than SOD, CAT, and ceruloplasmin [17]. MDA level in cattle with cutaneous papilloma was significantly higher than in healthy cattle [24]. In humans, high MDA levels have been detected in skin cancers such as basal cell carcinoma and non-melanoma skin carcinoma tissue that has been exposed to UV [39]. However, the available studies that describe blood antioxidant and lipid peroxidation status in skin diseases of cattle are few [40].

Our results revealed a significant decrease in the activity of GSH-Px and reduced GSH, confirming the oxidative action of IVM, which may lead to papillomas regression in cattle, besides SOD activity insignificantly decreased, and MDA activity insignificantly increased. As described in previous reports, IVM increased the production of ROS in leukemic cells [41]. IVM elicited ROS formation [42], causing damage to the cells, which may be eliminated either apoptosis or necrosis [43]. It was reported that IVM decreased plasma GSH concentrations without alterations in plasma MDA level leading to a decrease in the defense mechanism against oxidative stress, but this decrease is not enough to cause lipid peroxidation [44]. Similar results were reported with Bomectin^®^ (IVM) (data not published yet). Other studies recommended using a general tonic in IVM treatment due to its harmful effects on kidneys, hepatic functions, and oxidative stress [45].

Previous reports have suggested that the therapeutic effect of IVM against bovine skin warts is attributed to its cytotoxic effect on papilloma leading to a gradual loss in size and losing the vitality of wart mass [46] or its impact on the cellular immune response of the animal and showed a significant elevation in the rate of lymphocytic cells after IVM treatment [32]. The curative effect of IVM in the treatment of BP was previously documented, and it gave good results and recovery rates with a single or double dose [3,32]. Other studies have reported the antitumor [1] and immunostimulant [47] activities of IVM. The mechanism played by IVM as an antitumor agent is enhancing papilloma regression via inducing oxidative stress (ROS) [48,49].

## Conclusion

Finally, we can deduce that IVM’s immunostimulant and oxidative properties are responsible for the regression of bovine cutaneous papillomas. Further studies are needed to identify the antiviral activity of IVM against bovine papillomavirus. 

## List of abbreviations

PCV, packed cell volume; TLC, total leucocytes count; GSH-Px, glutathione peroxidase; IVM, ivermectin; BP, bovine papillomatosis; BPVs, bovine papillomavirus; MDA, malondialdehyde; GSH, glutathione reduced; SOD, superoxide dismutase; H&E, hematoxylin and eosin stain; ROS, reactive oxygen species; Hb, hemoglobin concentration; EDTA, Ethylenediaminetetraacetic acid; NADPH, Nicotinamide adenine dinucleotide phosphate; DTNB, Ellman’s Reagent; CAT, Catalase.
